# Dual Leap Motion Controller 2: A Robust Dataset for Multi-view Hand Pose Recognition

**DOI:** 10.1038/s41597-024-03968-9

**Published:** 2024-10-09

**Authors:** Manuel Gil-Martín, Marco Raoul Marini, Rubén San-Segundo, Luigi Cinque

**Affiliations:** 1 Grupo de Tecnología del Habla y Aprendizaje Automático (T.H.A.U. Group), Department of Electrical Engineering, Information Processing and Telecommunications Center, E.T.S.I. de Telecomunicación, Universidad Politécnica de Madrid, Madrid, Spain; 2 VisionLab, Department of Computer Science, Sapienza University, Rome, Italy

**Keywords:** Databases, Databases

## Abstract

This paper presents Multi-view Leap2 Hand Pose Dataset (ML2HP Dataset), a new dataset for hand pose recognition, captured using a multi-view recording setup with two Leap Motion Controller 2 devices. This dataset encompasses a diverse range of hand poses, recorded from different angles to ensure comprehensive coverage. The dataset includes real images with the associated precise and automatic hand properties, such as landmark coordinates, velocities, orientations, and finger widths. This dataset has been meticulously designed and curated to maintain a balance in terms of subjects, hand poses, and the usage of right or left hand, ensuring fairness and parity. The content includes 714,000 instances from 21 subjects of 17 different hand poses (including real images and 247 associated hand properties). The multi-view setup is necessary to mitigate hand occlusion phenomena, ensuring continuous tracking and pose estimation required in real human-computer interaction applications. This dataset contributes to advancing the field of multimodal hand pose recognition by providing a valuable resource for developing advanced artificial intelligence human computer interfaces.

## Background & Summary

Hand pose recognition based on computer vision is a very interesting component in the design of human-computer interfaces. This component allows identifying and understanding hand configurations and movements. This field can be framed within the broader context of Human Activity Recognition research^1,2^, where there has been substantial interest and exploration using vision-based approaches^3^, which aim to understand and interpret human actions and movements through visual data. In particular, hand pose recognition encompasses a diverse range of applications spanning from virtual reality and augmented reality to sign language recognition and gesture-based interfaces. At its core, hand pose recognition seeks to bridge the gap between human communication modalities and digital systems, enabling seamless interaction between users and technology through natural hand gestures and movements.

In the realm of virtual reality gaming, precise hand-tracking is crucial for achieving immersive gameplay experiences. For instance, certain games may require intricate hand poses or gestures to cast spells or manipulate virtual objects, requiring a level of accuracy that an isolated camera may struggle to achieve when a subject does not face their hands directly to the camera. Consequently, relying solely on single-view recording setups could potentially compromise the user experience. However, by implementing a multi-view recording setup featuring two cameras, developers can significantly enhance the capture of hand poses, mitigating issues such as occlusion and ensuring a more seamless interaction between players and the virtual environment.

Several notable datasets have been developed to advance the field of hand pose recognition^4^, presenting interesting aspects and some limitations. Most of those are focused on Sign Language Recognition tasks^5–7^, but others consist of general hand poses that could be used for human computer interaction applications. For example, Chen *et al*.^8^ provided a comprehensive resource with 320,000 synthetic images rendered from eight views using the TurboSquid ^a^ hand model. Similarly, Khaleghi *et al*.^9^ presented a dataset consisting of over 402,000 synthetic hand images captured from six different angles across ten distinct animated subjects. This dataset includes ground-truth 3D pose labels, which are instrumental in training robust 3D hand pose estimation models. However, while these datasets are particularly valuable for training models on multi-view hand pose estimation due to their great number of instances, they rely mostly on synthetic data. This dependence on synthetic imagery can limit the datasets applicability to real-world scenarios and may not fully capture the complexity and variability of real human hands. This important limitation impacts on the generalization capability of the models trained with this data.

Gomez-Donoso *et al*.^10^ presented a dataset which features over 20,500 instances, including annotated color images of hands from 9 subjects, with bounding boxes and the 2D and 3D positions of each joint. This dataset includes real images, and its annotations and multi-view setup provide a rich source for developing and evaluating hand pose recognition models. However, this dataset contains few instances and includes an artificial alternative set which comprises some of the hand poses with different random backgrounds.

The proposed dataset addresses these limitations by providing a multi-view dataset recorded using the Leap Motion Controller 2^11^, cutting-edge devices capable of directly obtaining precise hand landmarks^12^ in addition to capturing images. This dual capability allows for the creation of a richly detailed dataset that provides both comprehensive visual data and accurate landmark information, making it important for developing and testing advanced hand pose recognition systems. The use of this new device ensures that the dataset captures the fine nuances of hand movements and poses with unparalleled accuracy, further increasing its value for research and application in various technological fields. The dual perspective of the devices reduces ambiguities that might arise from a single viewpoint, allowing for more precise detection of hand poses by capturing details that may be obscured in one view but are visible in the other. This dataset offers a new opportunity for advanced hand pose recognition by leveraging information from both cameras, effectively addressing challenges such as angle variability and hand pose occlusion. Furthermore, the dataset’s versatility extends to scenarios where only a single camera viewpoint is available, enabling the evaluation of hand pose recognition systems under constrained conditions. This dual functionality not only enhances the robustness and adaptability of recognition models but also provides valuable insights into the performance trade-offs between multi-view and single-view approaches. The main contributions of this paper are the following:

- Presentation of Multi-view Leap2 Hand Pose Dataset (ML2HP Dataset), a new multi-view dataset. The paper presents the first dataset captured using a multi-view recording setup with Leap 2 devices, providing comprehensive coverage of hand poses from different angles.
- Use of Leap Motion Controller 2 for data acquisition. Leveraging the capabilities of this device, the dataset includes both images and precise automatic hand properties at frame level, enhancing the richness of the dataset.
- Balanced data variability in subjects, hand poses, and hand usage. The dataset encompasses a diverse range of subjects, hand poses, and hand usage (right or left), ensuring variability, balance, and richness in the recorded instances. This variability contributes to the robustness and generalization of hand pose recognition models trained on the dataset.

## Methods

This section describes the materials and recording setup used for the data collection, the data acquisition protocol, and the processing steps to curate the recorded data. In addition, a detailed description of the ML2HP dataset is also included.

### Materials and recording setup

This dataset has been recorded using two Leap Motion Controller 2 devices. These devices represent a significant advancement in hand-tracking technology, offering precise and responsive tracking capabilities essential for a wide range of digital and virtual applications. These devices are particularly suited for scenarios where capturing detailed hand poses is crucial, making it an ideal choice for research and development in human-computer interaction, virtual reality, and augmented reality environments.

The Leap Motion Controller 2 offers a substantial tracking range, detecting hand movements at depths ranging from 10 cm to 110 cm. These devices provide a wide 160° x 160° field of view, allowing a comprehensive tracking coverage within this range. The devices can capture hand movements at a maximum framerate of 115 frames per second. They have small dimensions of 84 mm × 20 mm x 12 mm (length x width x height) and a low weight of 29 grams. This compact form factor reduces obstructions and facilitates its incorporation in many setups.

**Fig. 1:**
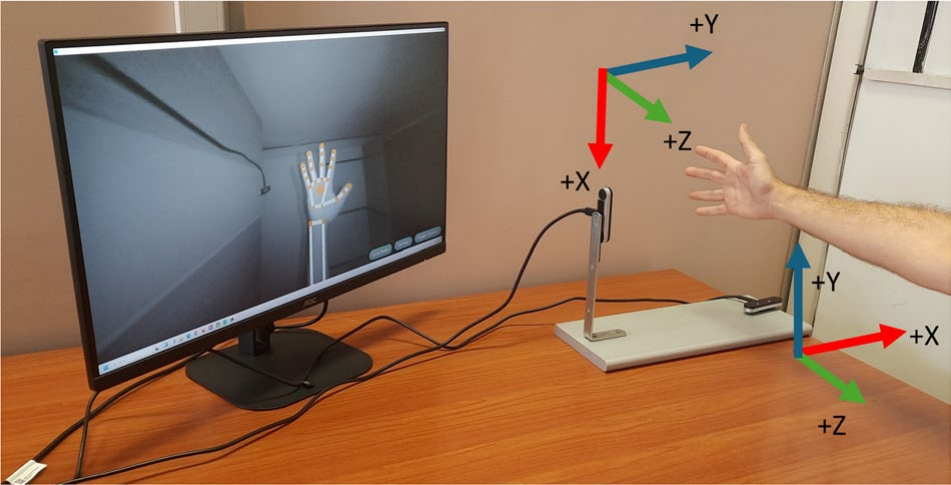
Example of data acquisition using both cameras placed on the platform detailing the coordinate systems.

**Fig. 2:**
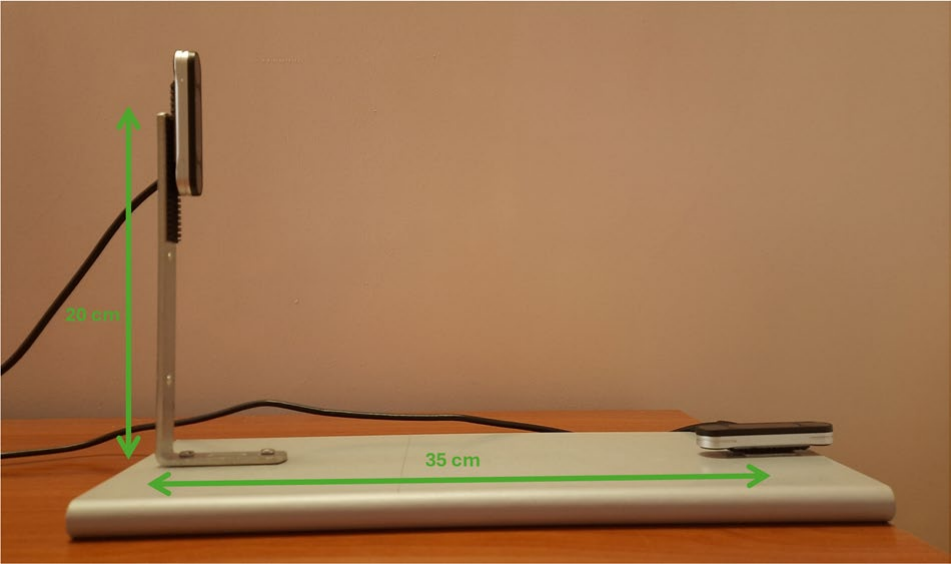
Horizontal and vertical distances between two cameras.

**Fig. 3:**
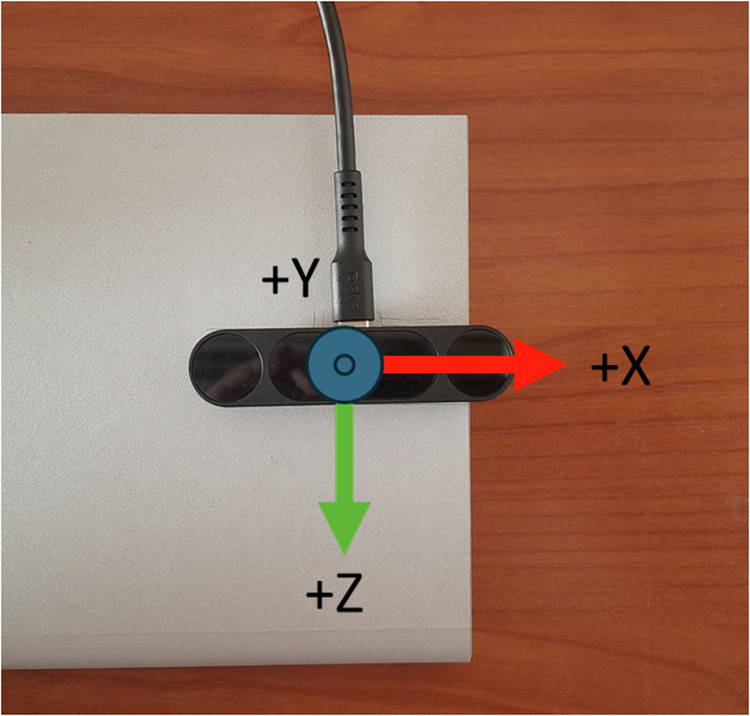
Right-handed Cartesian coordinate system of a Leap 2 Motion Controller.

For the data collection, the recording devices were connected to a high-performance laptop (Titan GT77 HX 13 V model) with the Ultraleap Gemini V5.2 software installed. The recording devices were placed on an ad hoc platform specifically designed to position them perpendicularly. Figure 1 shows an example of data acquisition using both cameras placed on the platform detailing the coordinate systems. This setup ensures that the devices can capture complementary perspectives of the hand poses. As shown in Fig. 2, there is a horizontal distance of 35 cm and a vertical distance of 20 cm between the two devices, optimizing the spatial coverage and accuracy of the recordings. This arrangement reduces the likelihood that any part of the hand will be hidden from both devices simultaneously. The devices use a right-handed Cartesian coordinate system, and the origin is centered at the top of the camera. The x- and z-axes lie in the plane of the camera sensors, with the x-axis running along the camera baseline. The y-axis is vertical, with positive values increasing upwards. The z-axis has positive values increasing toward the user. An example of the coordinate system is shown in Fig. 3.

### Data acquisition protocol

Before recording the data, each participant received detailed information about the data collection protocol and voluntarily provided informed consent, including their agreement to have the data published, by signing consent form prior to their inclusion in the research study. We contacted to the UPM ethics committee, but they informed us that official approval was not required and that the subjects’ consents were enough to collect and share the data. During the data collection process, participants were instructed to perform various hand poses while facing either one of the cameras, the other camera, or positioned diagonally between both. Additionally, participants were prompted to move their hands through the entire view range of the cameras, ensuring comprehensive coverage of hand poses from different angles, perspectives, and distances. This approach enabled the capture of a diverse range of hand configuration instances.

Participants were asked to perform each hand repeatedly pose until enough instances were recorded for each class and hand (right and left). The protocol involved initially recording instances of right-hand poses from all classes, repeating the process for left-hand poses. This systematic approach ensured comprehensive coverage of both right- and left-hand configurations across all classes, facilitating a balanced and representative dataset.

### Data curation and preparation

The software used in this study was built upon the open-source Native LeapcC API^13^, enabling seamless integration of Ultraleap’s Hand-tracking technology with C language. This software was adapted to simultaneously capture both images and hand properties from the hand-tracking system at specific timestamps using the MultiDeviceAware configuration of the Leap Connection.

To ensure the quality of the final dataset and avoid images where hands appear incomplete or at the borders of the images, we implemented a data curation process with specific threshold limits for hand landmark coordinates to remove undesirable locations (x and z coordinates must be in the interval [−350, 350] mm and y coordinates must be in the interval [70, 700] mm). This data curation was performed during the recording session: any image where the hand landmarks fall outside these defined limits was excluded from the dataset. This method helps to maintain the integrity of the images, ensuring that all included images display hands in their entirety and within the central region of the frame.

Additionally, to avoid redundancy, we removed instances containing the same information by filtering out images where the palm position coordinates remained stable. Specifically, if the x, y, and z coordinates of the palm position differed by less than 3 mm between any pair of frames in a hand pose acquisition, only one instance was retained. This step ensures that the dataset contains diverse and informative examples but also a high number of instances captured.

Given the variability in the sampling frequency of the cameras, the software employed a synchronization mechanism to align the available instances from both devices based on their respective timestamps. Instances were saved based on strict exclusion criteria, ensuring that only instances where both images and hand properties were successfully obtained at a specific timestamp were saved for the dataset. The software ensured accurate synchronization of the data despite the unstable sampling frequencies of the cameras. This synchronization approach is essential for subsequent analysis and interpretation of the recorded hand poses from different points of view. Thorough experimentation, we determined that a threshold of ten milliseconds was effective in ensuring that two frames from different cameras could be considered as corresponding to the same instant in time.

All these data curation procedures were performed during the recording session, which allows us to record a great number of instances and balance the dataset.

Finally, from all the obtained instances per subject, hand used, and pose, we selected 1,000 random samples from the available data to include samples of hand facing the horizontal device, the vertical device or both. To maintain reproducibility and avoid losing synchronization, we used a fixed seed during the random selection process. This approach ensures that given the same data length, the same instances are selected each time for both devices, preserving the consistency of the dataset.

In addition, the Ultraleap software independently detected the width of the metacarpal, proximal, intermediate, and distal phalanges of each finger. However, our observations indicated that the widths of all the phalanges of the same finger were consistent. This way, we decided to retain a single general width measurement per finger and remove the redundant width data for each phalange. This cleaning process reduced unnecessary data redundancy while maintaining the data integrity of the measurements.

### Dataset overview

The ML2HP Dataset encompasses a rich set of hand poses recorded with two Leap Motion Controller devices simultaneously. These devices provide images and a wide variety of hand information, including position, velocity, and orientation from different hand key points and finger widths. The dataset contains 714,000 instances from 21 subjects performing 17 different hand poses from the right and the left hand.

The dataset has been meticulously recorded and curated to maintain a balance in terms of hand poses and subjects, distinguishing between right and left hands. This balanced distribution not only fosters fairness in the dataset but also ensures that any subsequent analysis or model training is not biased towards any specific hand pose, subject, or hand predominance.

**Fig. 4:**
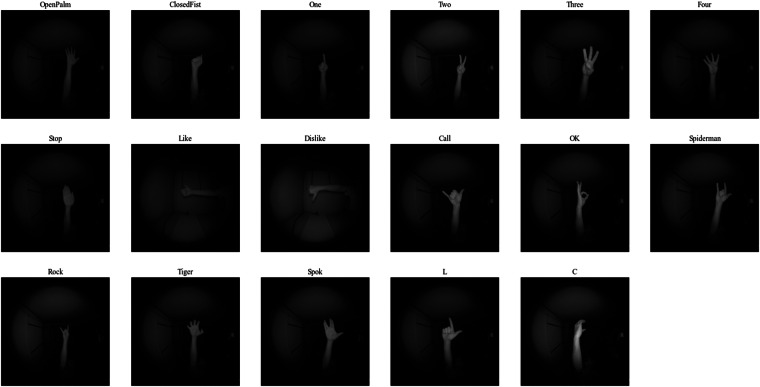
Samples of each hand pose.

Participants were informed about the specific hand poses to be performed before the data collection process to ensure accurate and consistent recordings. The selected hand poses were chosen based on their prevalence in existing datasets related to single-hand interactions, ensuring relevance and compatibility with real-world applications like gaming. Moreover, less common hand poses were also included to enrich the hand pose variability. Their extensive range and diversity enable a nuanced approach to hand pose recognition, contributing to the dataset’s complexity. This pose variability also introduces challenges for machine learning algorithms, particularly in scenarios where hand orientation to the camera may cause poses to appear similar, enhancing the dataset’s utility for complex and advancing hand pose recognition systems. The dataset comprises 17 distinct hand poses, each named and described as follows. Figure 4 shows one example per class.

- **Open Palm**, where all fingers are extended and spread apart.
- **Closed Fist**, with all fingers clenched into the palm.
- **One**, where only the index finger is extended with the other fingers clenched.
- **Two**, with the index and middle fingers extended together and the remaining fingers clenched.
- **Three**, where the index, middle, and ring fingers are extended together while the pinky and thumb are clenched.
- **Four**, with all fingers except the thumb extended and held together.
- **Stop**, involving the hand held with the palm facing outward and all fingers extended together, resembling a stop gesture.
- **Like**, featuring the thumb extended upward with the other fingers clenched into a fist.
- **Dislike**, with the thumb extended downward and the other fingers clenched into a fist.
- **Call**, where the thumb and pinky fingers are extended.
- **OK Sign**, with the thumb and index finger forming a circle while the other fingers are extended.
- **Spiderman**, where the fist is clenched while the thumb, index and pinky are extended.
- **Rock**, where the fist is clenched while the index and pinky are extended.
- **Tiger**, with all fingers extended and slightly curled, emphasizing the nails.
- **Spok**, with the middle and ring fingers separated, creating a “V” shape, while the thumb is extended away from the palm.
- **L**, where the thumb and index finger are extended perpendicular to each other forming an L shape.
- **C**, with the thumb and rest of fingers curved to form a C shape.

The dataset comprises information from a diverse group of participants, totaling 21 individuals (17 males and 4 females), whose ages range across the adult spectrum (22-68 years old). Before recording pose instances, participants underwent an assessment to capture demographic information (age and gender). Table 1 summarizes the demographic information from the subjects of the dataset. Since a potential target application scenario was focused on gameplay, we selected participants around their twenties, as this age group is a common target for gaming.

**Table 1:** Demographic information from the subjects of the dataset.

Subject ID	Age	Gender
001	30	Male
002	25	Male
003	26	Male
004	27	Male
005	27	Male
006	27	Male
007	26	Male
008	25	Male
009	29	Male
010	32	Male
011	23	Female
012	24	Male
013	25	Male
014	27	Male
015	27	Female
016	26	Female
017	34	Male
018	28	Female
019	22	Male
020	68	Male
021	22	Male

## Data Records

**Fig. 5:**
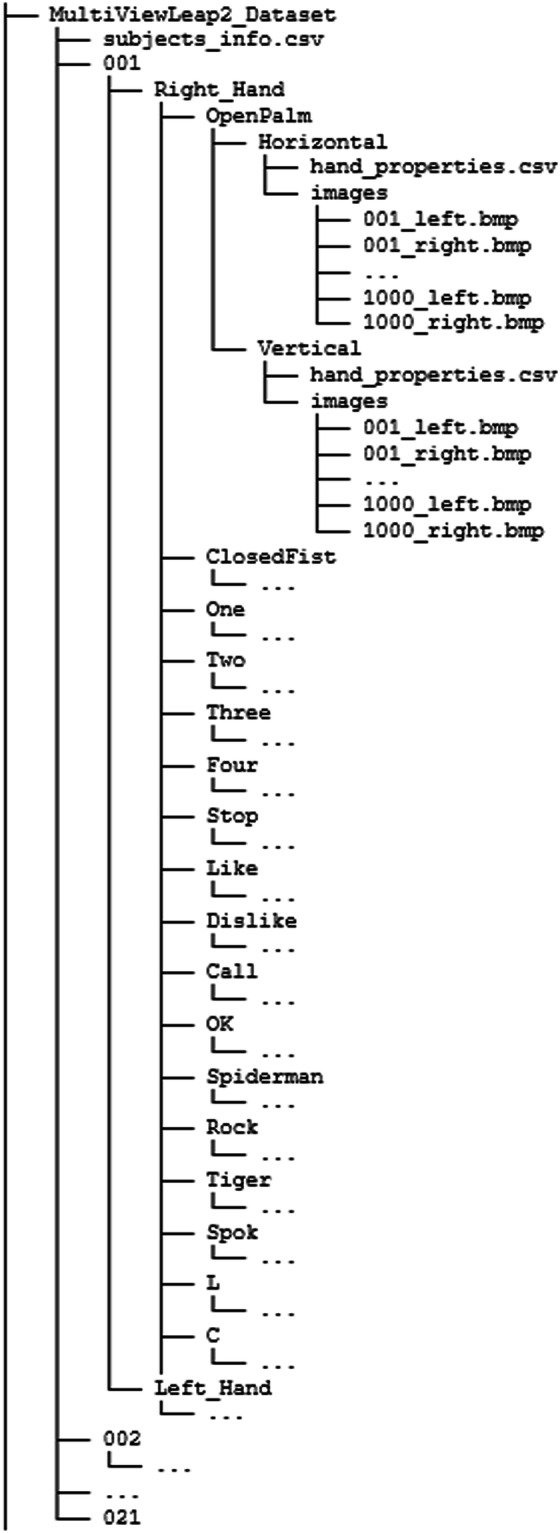
Hierarchical file structure of the dataset.

The ML2HP Dataset is available at e-cienciaDatos repository^14^. It is meticulously organized into a hierarchical file structure to facilitate easy access and retrieval of specific instances for analysis. Figure 5 shows the file structure of the dataset obtained once each.zip file of the repository corresponding to each subject is unzipped. At the top level, there is a folder for each subject, identified by integer numbers (e.g., “001”, “002”). Within each subject folder, there are subfolders representing the hand used, designated as “Right_Hand” and “Left_hand”. Inside each hand folder, further subfolders are categorized by hand pose class, named according to the specific hand pose (e.g., “OpenPalm”, “ClosedFist”, etc). Each pose class folder contains two additional subfolders corresponding to the recording devices: “Horizontal” for the camera placed horizontally and “Vertical” for the camera placed vertically. In addition, at the top level, there is a “subjects_info.csv” file that includes the information of age and gender for each subject identifier.

Within each folder associated to each device, there exists an “images” subfolder that contains the images corresponding to the 1,000 instances: a total of 2,000 images in “.bmp” format, capturing the hand pose from the respective device perspective. Specifically, these images are divided evenly, with 1,000 captured from the left camera (files finishing in “_left.bmp”) and 1,000 from the right camera (files finishing in “_right.bmp”) of the stereo camera setup in the Leap Motion Controller 2. Within this device-specific folder there is also a single “hand_properties.csv” file that contains detailed information about the hand: automatic hand properties such as landmarks data or finger widths, and other relevant data for each image. The LeapC API uses specific units to ensure precise and standardized data collection for the different physical quantities. Distances are recorded in millimeters, speed is quantified in millimeters per second, and angles are measured in radians. In particular, the “.csv” file contains 247 columns, which correspond to the following information:

- Basic information (5 columns):
  - frame_id: Identifier for the frame from a particular subject, hand and pose are being recorded.
  - subject_id: Identifier for the individual whose hand and pose are being recorded.
  - which_hand: It indicates which hand (Left_Hand or Right_Hand) is being measured in the dataset.
  - pose: Name that represents the pose of the hand.
  - device: Identifier for the specific device used to capture the data (Horizontal or Vertical).
- Pinch and Grab Metrics (4 columns):
  - pinch_distance: Distance between index and thumb finger, measured in millimeters.
  - grab_angle: Average angle of fingers to palm. It could assume values from 0 to π, where 0 means fully open and π fully closed, simulating the radians.
  - pinch_strength: The normalized estimate of the pinch pose (a pinch is between the thumb and any other finger. This property has values in the interval [0, 1], where zero is not pinching; one is fully pinched.
  - grab_strength: The normalized estimate of the grab hand pose (a grab is all four fingers (excluding thumb) curled towards the palm). This property has values in the interval [0, 1], where zero is not grabbing; one is fully grabbing.
- Palm Position, Velocity, Normal, and Orientation (17 columns):
  - palm_width: Width of the palm, measured in millimeters.
  - palm_position_x, palm_position_y, palm_position_z: Coordinates of the palm’s position in a 3D space, measured in millimeters.
  - palm_velocity_x, palm_velocity_y, palm_velocity_z: Velocity of the palm’s movement in each direction, measured in millimeters/second.
  - palm_normal_x, palm_normal_y, palm_normal_z: Normal vector to the palm. If the hand is flat, this vector will point downward, or “out” of the front surface of your palm. These properties could have values in the interval [−1, 1]. The direction is expressed as a unit vector pointing in the same direction as the palm normal (that is, a vector orthogonal to the palm).
  - palm_direction_x, palm_direction_y, palm_direction_z: Direction vector of the palm position toward the fingers. These properties could have values in the interval [−1, 1]. The direction is expressed as a unit vector pointing in the same direction as the directed line from the palm position to the fingers.
  - palm_orientation_x, palm_orientation_y, palm_orientation_z, palm_orientation_w: Orientation of the palm using quaternion notation. These properties could have values in the interval [−1, 1] radians.
- Fingers General Information: for each finger (thumb, index, middle, ring and pinky) the following general characteristics are available (2 × 5 = 10 columns):
  - width: The thickness of the finger segment, measured in millimeters.
  - is_extended: A Boolean value indicating whether the finger segment is extended (straight) or not.
- Fingers Specific Information: for each finger (thumb, index, middle, ring, and pinky) the following specific characteristics for each phalange (metacarpal, proximal, intermediate, and distal) are available, including their positions and rotations (10 × 5 x 4 = 200 columns):
  - prev_joint_x, prev_joint_y, prev_joint_z: 3D coordinates (x, y, z) of the previous joint (closer to the heart) of the finger phalange segment, measured in millimeters.
  - next_joint_x, next_joint_y, next_joint_z: 3D coordinates (x, y, z) of the next joint (further from the heart) of the finger phalange segment, measured in millimeters.
  - rotation_x, rotation_y, rotation_z, rotation_w: Rotation in world space from the forward direction of the finger phalange segment. These properties could have values in the interval [−1, 1] radians.
- Arm Metrics (11 columns):
  - arm_width: Width of the arm, measured in millimeters.
  - arm_prev_joint_x, arm_prev_joint_y, arm_prev_joint_z: 3D coordinates (x, y, z) of the previous joint of the arm (closer to the heart), measured in millimeters.
  - arm_next_joint_x, arm_next_joint_y, arm_next_joint_z: 3D coordinates (x, y, z) of the next joint of the arm (further to the heart), measured in millimeters.
  - arm_rotation_x, arm_rotation_y, arm_rotation_z, arm_rotation_w: Rotation in world space from the forward direction of the arm. These properties could have values in the interval [−1, 1] radians.

**Fig. 6:**
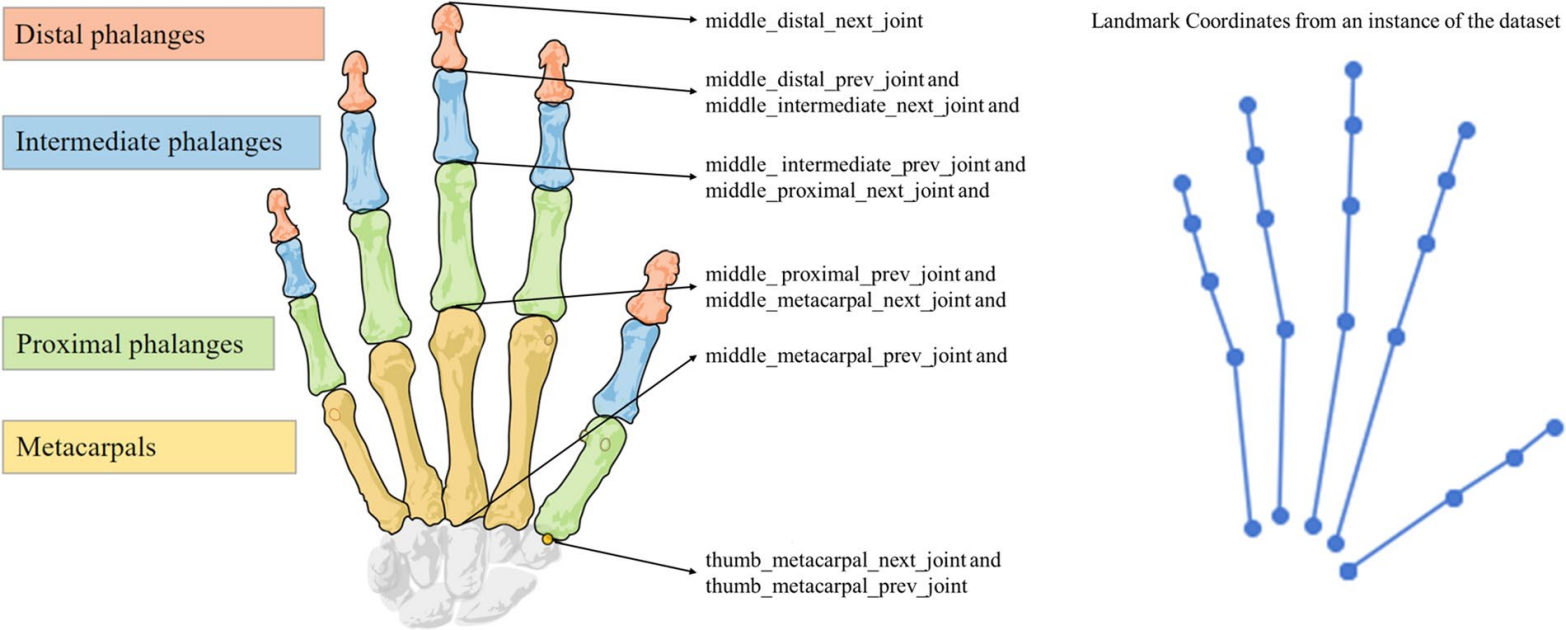
Information about finger joints and an example of the finger landmark coordinates.

In the context of finger joint data, it is reasonable to assert that the “next_joint_*” coordinates of a phalange correspond to the “prev_joint_*” coordinates of the subsequent phalange. This relationship maintains a continuous and sequential connection along the finger’s anatomy, ensuring that the endpoint of one segment matches the starting point of the next. Even though these landmarks have the same values, they are kept in the dataset to better understand the phalanges. This facilitates easier computation of phalange lengths and provides a more intuitive representation of finger anatomy. Additionally, for the thumb metacarpal, the “prev_joint_*” and “next_joint_*” coordinates are the same, reflecting the unique structure of the thumb where the metacarpal bone connects directly to the wrist and only has one joint connection point, unlike the other fingers which have multiple phalanges. Figure 6 displays this information about the finger joints in a diagram and an example of the finger landmark coordinates.

This structured organization ensures that each subject’s hand poses are systematically categorized and easily accessible, facilitating comprehensive and efficient analysis. This meticulous arrangement supports the development and validation of robust hand pose recognition models by providing a well-organized and richly detailed dataset.

## Technical Validation

This section presents analyses performed to support the dataset’s technical quality and justify the data’s reliability.

**Fig. 7:**
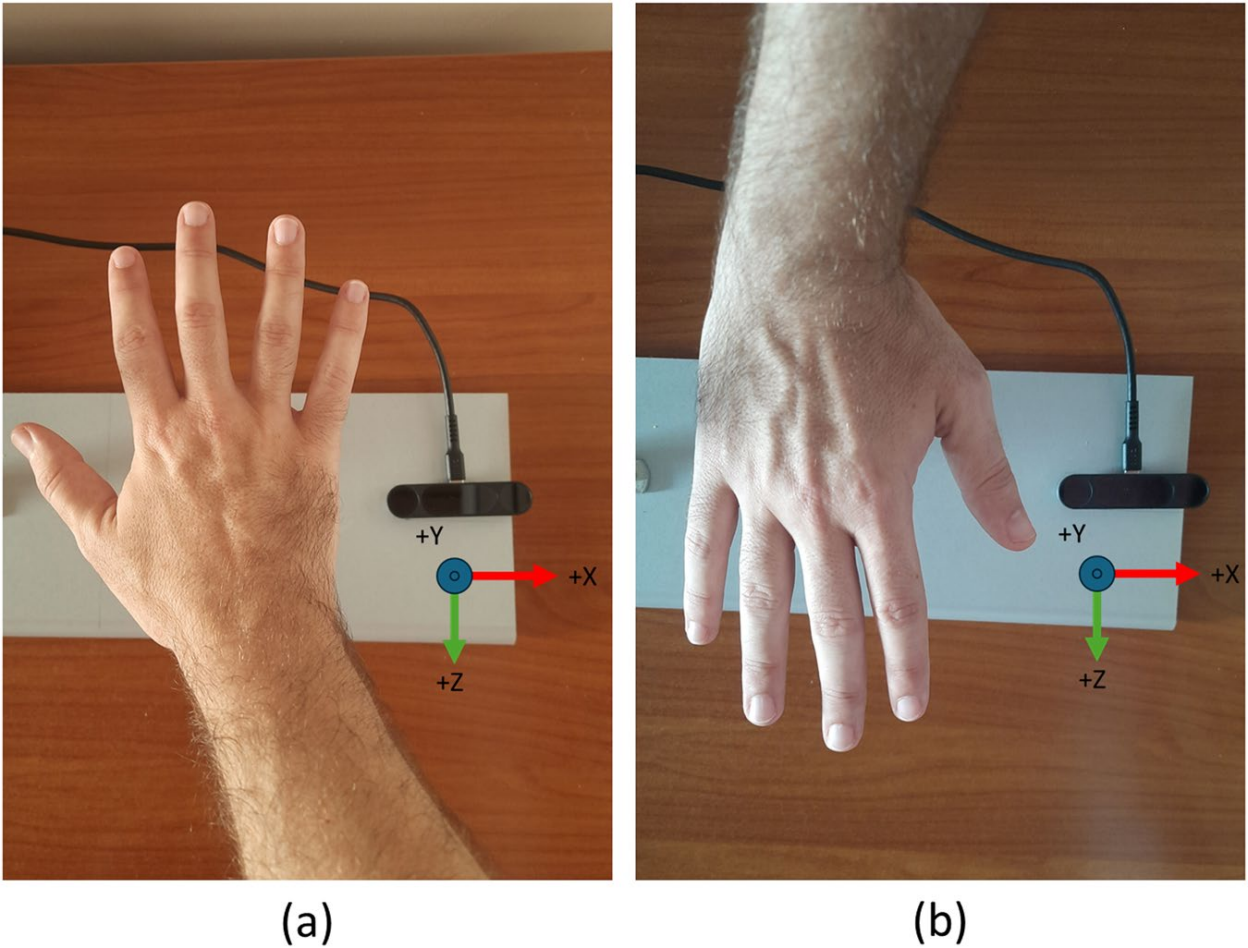
Correct (**a**) and unadvisable (**b**) hand positioning for proper tracking.

Before the data collection process, we conducted various experiments to determine the optimal orientation of the cameras during recording. One setup that proved to be highly unadvisable involved positioning the Leap Motion Controller 2 devices such that the palm of the hand faced the camera, with the wrist aligned along the -Z axis and the fingers pointing towards the +Z axis, as shown in Fig. 7(b). In this configuration, the device encountered significant difficulties tracking the information on hand properties. Consequently, this setup was deemed unsuitable for reliable data collection and was avoided in our final protocol. This way, we used a hand positioning with the fingers pointing towards the -Z axis as shown in Fig. 7(a). In terms of the positioning, in the beginning, we placed the cameras closer (with a horizontal distance of 25 cm to each other), but we realized that this way, the hand was too close to the vertical device when it was placed in the middle of the horizontal device (location from which the subjects started the data collection). This way, we decided to place the cameras with a horizontal distance of 35 cm and a vertical distance of 20 cm between the two devices to optimize spatial coverage. This way we could ensure the capture of instances that were close to both cameras, close to one camera and far from the other, and vice versa.

Another critical aspect of our technical validation during the data collection protocol addressed the challenge posed by the unstable sampling frequency of the cameras. This issue required us to collect data from both cameras independently and subsequently synchronize the final dataset. To achieve this, we conducted preliminary experiments to select the appropriate frames from each camera that corresponded to the same moment in time. We initially tested two available codes that integrate Ultraleap Tracking Hands functionality: the Gemini LeapC Python Bindings^15^ and the Native LeapcC API^13^. After analyzing both possibilities, we observed that the Python bindings employed a scheduler based on listeners, significantly delaying data collection and excessively decreasing the sampling rate. Ultimately, we decided to use the Native LeapC API in C language code, combined with a synchronization strategy, to align the data accurately. As mentioned above, we empirically determined that a threshold of ten milliseconds effectively ensured that two frames from different cameras could be considered as corresponding to the same instant in time. We also experimented with increasing the threshold, keeping the horizontal-vertical instance pairs with the closest timestamps and removing the rest. However, increasing the threshold led to a lower number of valid instances as it caused the unnecessary removal of valid instances. Thus, a ten-millisecond threshold was found to be the most effective threshold for maintaining synchronization between series with 100 frames per second, without losing significant data. This meticulous synchronization process was essential to maintain the integrity and temporal consistency of the dataset, thereby enhancing its reliability and utility for hand pose recognition tasks.

**Fig. 8:**
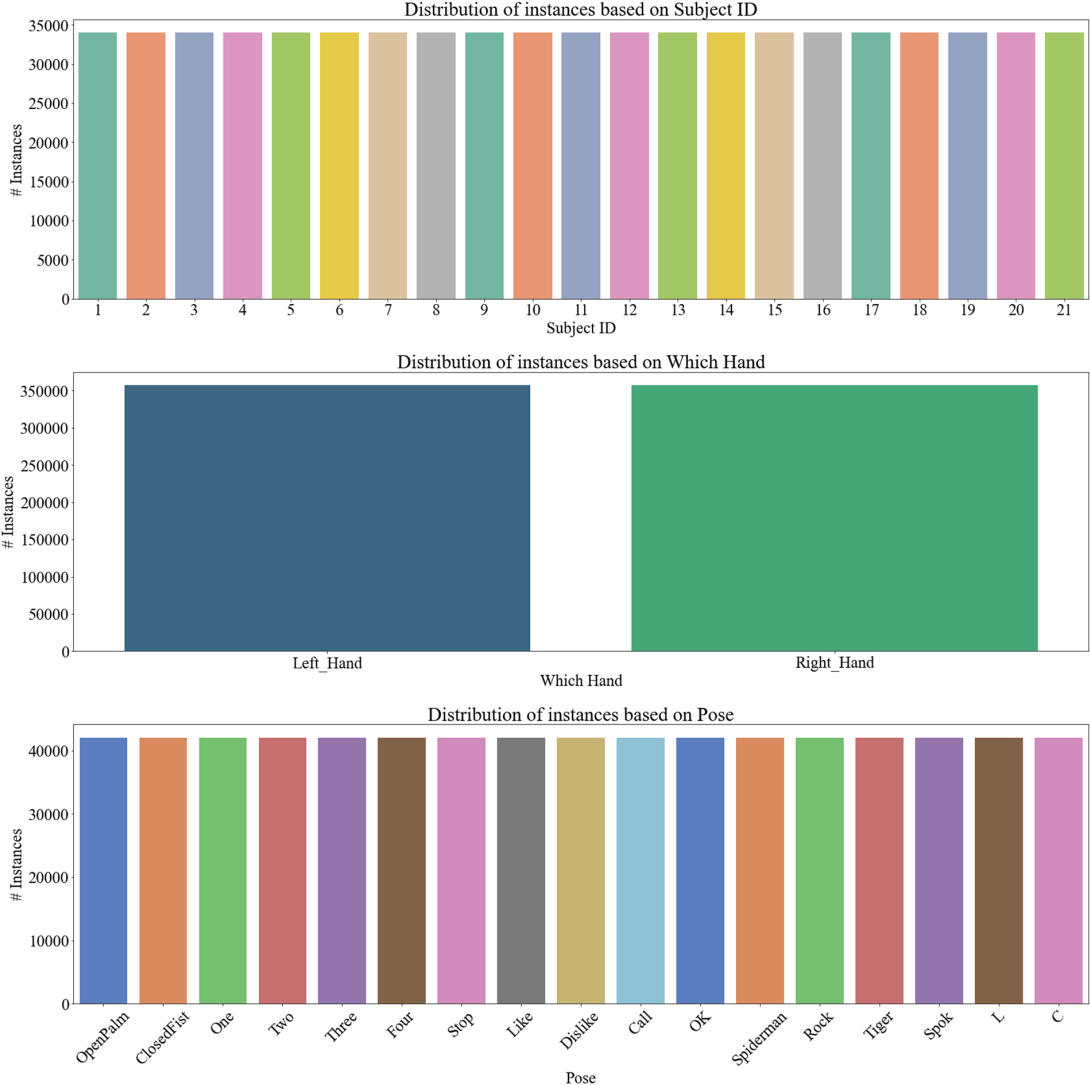
Distribution of instances per subject, hand, and pose.

Among the technical validation procedures employed to ensure the quality and reliability of this dataset, we have rigorously checked for potential biases related to subjects, right/left-hand usage, and hand poses during the data collection protocol. The resulting data has been processed to analyze the distribution of the final instances across different subjects, ensuring that each hand (right and left) and each hand pose class are equally represented. Figure 8 illustrates the total number of instances (714,000) distributed among various subjects (34,000 per subject) and the balance between the right and left hands (357,000 per hand) and the different hand pose classes (42,000 per pose). It is fair it say that for each instance, there is information from horizontal and vertical devices (including stereo images and hand properties). This thorough validation confirms that the dataset is balanced and free from these biases, thereby supporting the careful data collection protocol and curation procedures followed and the robustness and utility of the proposed dataset for developing and evaluating hand pose recognition models.

**Fig. 9:**
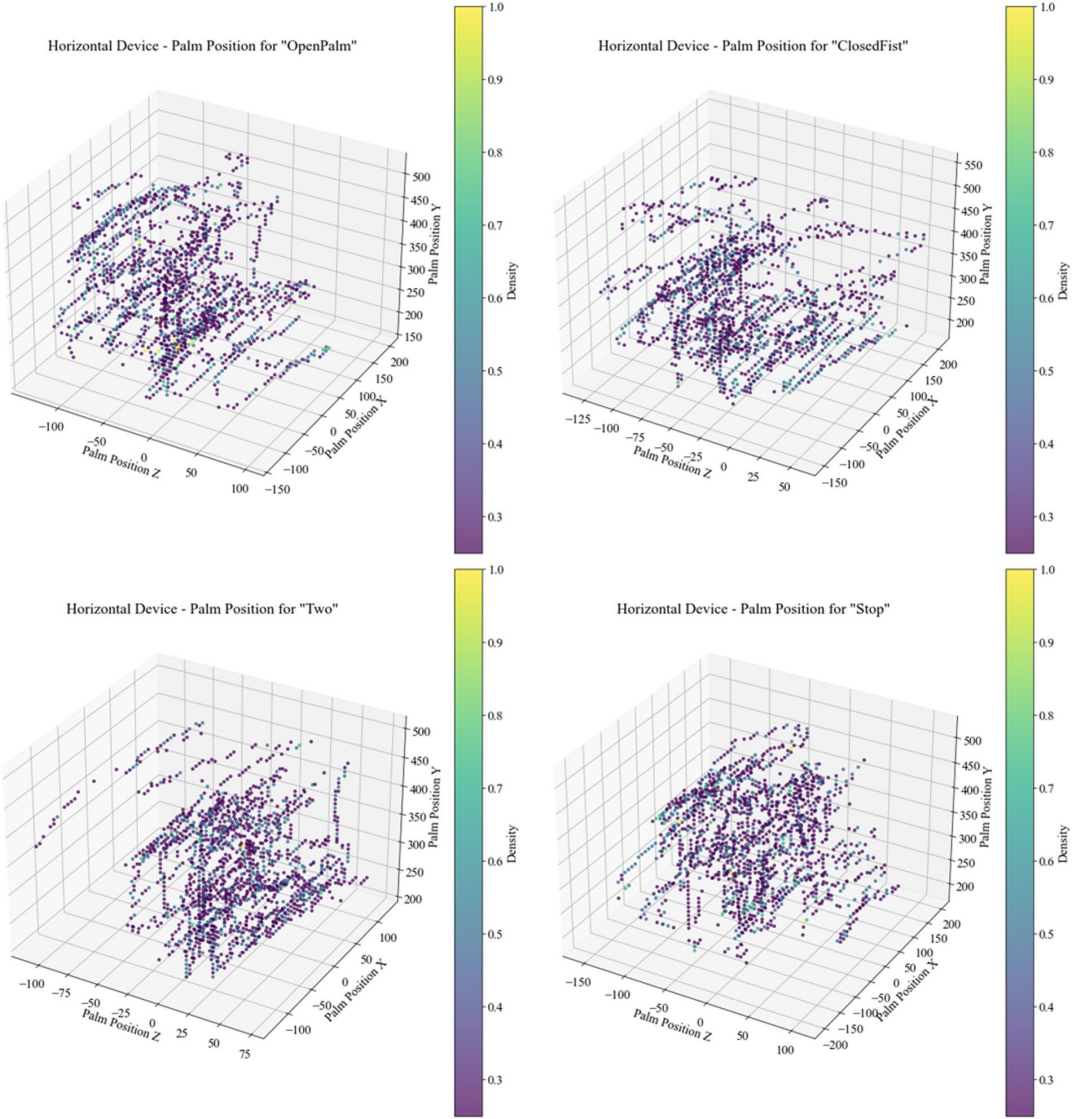
Scattering representation of hand center coordinates for some classes using the horizontal device.

As part of our technical validation procedure, we conducted an in-depth analysis of the dataset to assess the variability of hand locations within the spatial domain. To illustrate the dataset, the following information is related to subject 001. We used the palm center coordinates obtained from both cameras to accomplish the variability of hand locations. This analysis aimed to ensure that the dataset encompasses a diverse range of hand positions, critical for training robust hand pose recognition models. Figure 9 depicts scatter plots for some classes separately, showcasing the spatial distribution of hand positions for the Horizontal device. It is fair to state that the Vertical device perspective would show a similar distribution of the palm position scattering in the space since the devices are faced to a specific area where the hand poses have been collected. This figure shows a comprehensive view of this variability by presenting scatter plots derived from the data captured, offering insights into the overall dataset quality and coverage. These aspects enhance the dataset utility for training and evaluation purposes.

**Fig. 10:**
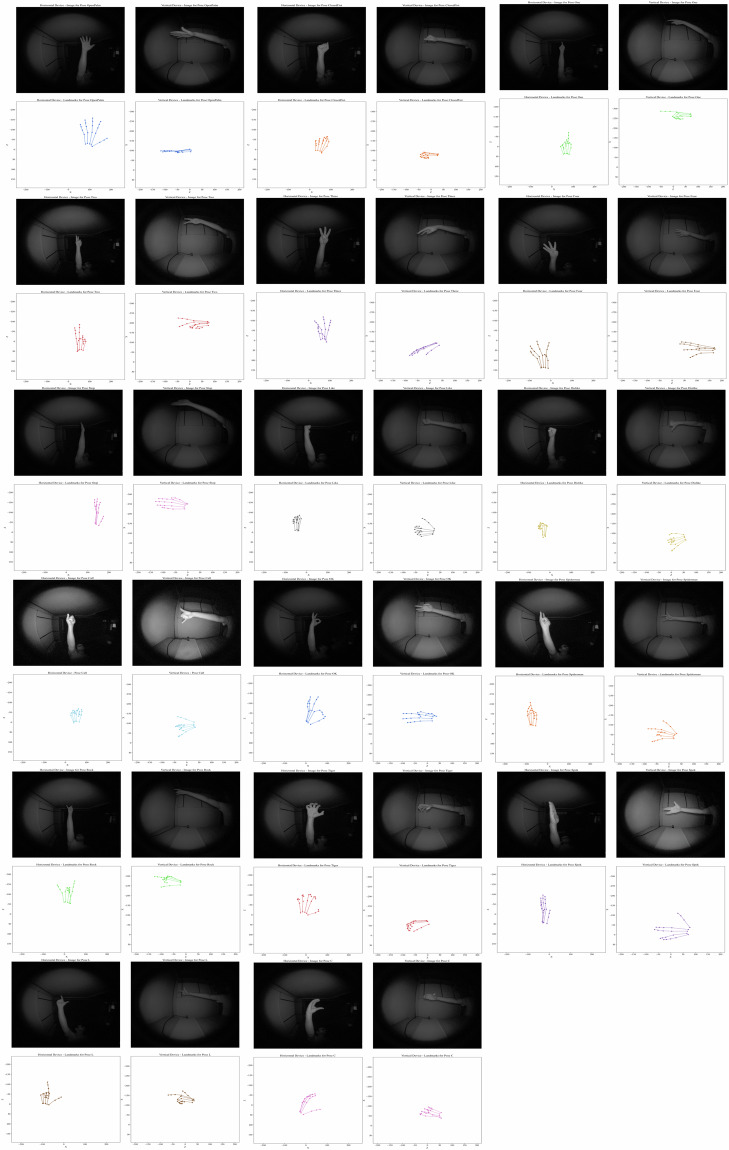
Images and 2D landmark representations for examples from the different classes using both devices.

To illustrate with some instances of the dataset, Fig. 10 represents the images from Horizontal and Vertical devices alongside their corresponding 2D landmark representations, offering a visual correlation between the raw image data and the Leap2 obtained landmark coordinates. This approach allowed us to directly observe the available points of view and the consistency of the finger joint landmarks.

In this illustration analysis, it is possible to observe several instances where the orientation of the hand relative to the devices could impact the hand pose detection when using an isolated device. When the hand was directly faced toward the horizontal device, the vertical device often struggled to capture the hand pose correctly due to occlusion and limited visibility of the fingers, as shown in the examples related to Open Palm, Three, OK, Tiger or C hand poses of the figure. Conversely, when the hand was oriented directly towards the vertical device, the horizontal device encountered similar challenges, leading to uncomplete landmark representation, as shown in the examples related to Stop, Like, Dislike, Call, Spiderman or Spok hand poses of the figure. However, in some scenarios where the hand was positioned diagonally, both devices were able to inform perfectly about the hand pose, as shown in the examples related to Two, Four, Rock or L hand poses of the figure. The diagonal orientation provided a more comprehensive view of the hand’s geometry to both devices, minimizing occlusions and allowing for more precise and consistent landmark detection across different hand poses. Moreover, there are some hand poses that due to its nature, they could be easily detected from both devices independently of the hand orientation, as shown in the examples related to Closed Fist or One hand poses of the figure. This way, the dual-view setup could effectively mitigate the occlusion issues and enhance the overall hand pose classification performance. Complementing the information from both devices in challenging instances could overcome the limitations inherent in a single-view setup^16^.

**Fig. 11:**
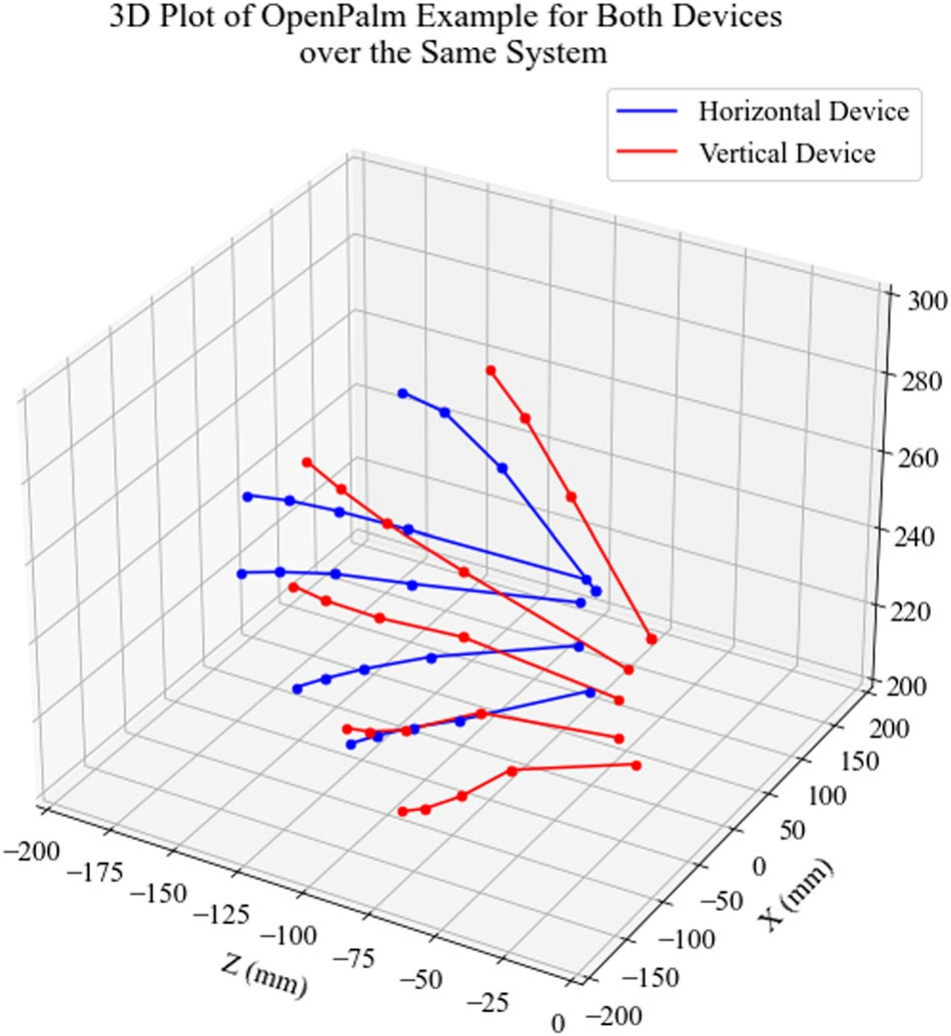
3D Plot of OpenPalm Example for Both Devices over the Same Coordinate System.

The accuracy of the hand pose estimation was quantitatively assessed by calculating the mean and standard deviation of the absolute errors in the x, y, and z coordinates of fingers obtained from Horizontal and Vertical devices across multiple subjects, hands, and poses. These errors were computed by transforming the coordinate system of one camera view to the other and then comparing the coordinates. As an illustration, Fig. 11 shows a 3D plot of an OpenPalm example for both devices over the same coordinate system. In this example, the error was 11.97 ± 6.79 mm (mean ± std), with 21.13 mm and 3.58 mm maximum and minimum errors, respectively.

However, higher mean errors were observed in instances where occlusions occurred, suggesting challenges in accurately capturing joint positions in such scenarios. For example, the aggregated mean and standard deviation errors for each hand and pose combination from subject 001 are summarized in Table 2. The results indicate consistent performance across different configurations because the mean error between the landmark coordinates from both cameras is around 21 ± 12 mm (mean ± std). This table also informs about the maximum and minimum error obtained in the estimation between the devices, reaching maximum values around 112 mm and minimum values around 10^−4^ mm.

**Table 2:** Mean, standard deviation, maximum and minimum errors between the hand pose estimation from Horizontal and Vertical devices.

Hand	Pose	MeanError (mm)	StdError (mm)	MaxError (mm)	MinError (mm)
Left	OpenPalm	17.95	10.24	103.30	1.50 · 10^−5^
Left	ClosedFist	22.25	12.71	94.55	5.68 · 10^−14^
Left	One	21.22	11.91	92.28	5.95 · 10^−4^
Left	Two	20.57	11.54	102.29	1.42 · 10^−3^
Left	Three	20.80	9.56	80.40	7.30 · 10^−5^
Left	Four	22.48	11.01	120.10	2.44· 10^−4^
Left	Stop	20.52	9.58	105.86	2.75 · 10^−3^
Left	Like	18.05	10.21	62.06	8.39· 10^−4^
Left	Dislike	19.59	11.34	114.59	1.50 · 10^−5^
Left	Call	21.30	12.81	137.47	5.80· 10^−4^
Left	OK	20.31	10.43	116.13	2.59· 10^−4^
Left	Spiderman	23.15	13.17	134.32	9.10 · 10^−5^
Left	Rock	16.46	9.45	100.34	1.64· 10^−4^
Left	Tiger	17.66	9.39	92.28	7.60 · 10^−5^
Left	Spok	15.83	9.96	96.51	9.20 · 10^−5^
Left	L	22.42	11.57	124.41	1.41 · 10^−3^
Left	C	19.75	11.46	91.81	9.20 · 10^−5^
Right	OpenPalm	20.09	11.07	163.34	4.93 · 10^−4^
Right	ClosedFist	21.69	13.86	86.74	6.12 · 10^−4^
Right	One	21.57	13.00	119.95	8.85 · 10^−4^
Right	Two	20.73	12.42	91.00	5.09 · 10^−4^
Right	Three	23.10	13.85	94.96	5.68 · 10^−14^
Right	Four	21.62	13.03	126.13	4.42 · 10^−4^
Right	Stop	20.58	12.82	109.56	1.53 · 10^−4^
Right	Like	20.67	12.47	120.43	1.74 · 10^−3^
Right	Dislike	23.36	14.10	130.36	3.51 · 10^−4^
Right	Call	22.86	15.05	140.78	3.23 · 10^−3^
Right	OK	17.52	10.90	96.76	3.20 · 10^−4^
Right	Spiderman	23.26	15.22	119.83	4.20 · 10^−5^
Right	Rock	21.81	14.56	108.16	1.60 · 10^−4^
Right	Tiger	24.66	17.72	119.97	9.00 · 10^−4^
Right	Spok	18.27	11.72	188.92	7.33 · 10^−4^
Right	L	21.67	13.05	112.39	6.56 · 10^−4^
Right	C	19.70	13.70	96.57	1.68 · 10^−4^

## Data Availability

The functions used for the data collection are from the open source Native LeapC API: https://docs.ultraleap.com/api-reference/tracking-api/index.html.
